# Choroidal thickness as a biomarker of inflammatory activity in patients with polymyalgia rheumatica receiving anti–IL-6 treatment

**DOI:** 10.3389/fmed.2026.1917539

**Published:** 2026-08-17

**Authors:** Laura Trives-Folguera, Santiago Muñoz-Fernández, María del Mar Esteban-Ortega, Javier Leyva-Esteban, Raquel Coca-Serrano, Tatiana Cobo-Ibañez, Cristina Vergara-Dangond, Liz Romero-Bogado, Isabel de la Cámara-Fernández, Patricia Richi, María Beatriz Paredes, Ana Esteban-Vazquez, Ana Valeria Acosta, Gabriela Cueva-Najera, Marco Algarra-San José, Israel Thuissard-Vasallo, Martina Steiner

**Affiliations:** 1 Department of Rheumatology, Hospital Universitario Infanta Sofía, Madrid, Spain; 2 Department of Medicine, Faculty of Medicine, Health and Sports, Universidad Europea de Madrid, Madrid, Spain; 3 FIIB HUIS-HUHEN, Madrid, Spain; 4 Department of Ophthalmology, Hospital Universitario Infanta Sofía, Madrid, Spain

**Keywords:** choroidal thickness, C-reactive protein, inflammation, interleukin-6, polymyalgia rheumatica

## Abstract

**Objective:**

In corticosteroid-dependent polymyalgia rheumatica (PMR), anti–interleukin-6 (IL-6) therapies are frequently used; however, they may limit the reliability of acute-phase reactants (APRs) as biomarkers of disease activity. Choroidal thickness (CT) may serve as an alternative inflammatory biomarker in these patients. So, the aim of this study is to evaluate changes in CT in patients with PMR initiating anti–IL-6 therapy over 6 months follow-up period, and to assess its correlation and level of agreement with other inflammatory parameters.

**Methods:**

It is a prospective, observational, longitudinal pilot study. We included 24 patients with PMR who initiated anti–IL-6 therapy. Assessments were performed at baseline (treatment initiation), and at 1 and 6 months. Each visit included a physical examination, musculoskeletal ultrasound of the shoulders and hips, blood tests (including C-reactive protein [CRP]), and CT measurement by optical coherence tomography (OCT). Disease activity was assessed using the PMR Activity Score (PMR-AS) and its imputed version (PMR-ASimp).

**Results:**

Mean baseline CT was 214 ± 71.13 μm. CT decreased significantly at 1 and 6 months after treatment initiation (208.46 ± 70.03 μm, *p* < 0.001 and 183.67 ± 70.37 μm, *p* < 0.001, respectively). A significant correlation was observed between the reduction in CT and the decrease in prednisone dose from 1 to 6 months (*r* = 0.417, *p* = 0.043). No significant correlations were found between CT and the other assessed parameters. Agreement between changes in CT and CRP was 83.3% at 1 month and 79.1% at 6 months. Agreement between changes in CT and PMR indices was 83.3% at both 1 and 6 months.

**Conclusion:**

In patients with active PMR, CT was increased at baseline and decreased significantly following initiation of anti–IL-6 therapy. Agreement between changes in CT and changes in CRP and PMR indices was high. These findings suggest that CT may be useful as a potential inflammatory biomarker in PMR, although further studies are needed to confirm this hypothesis.

## 1 Introduction

Polymyalgia rheumatica (PMR) is the most common inflammatory rheumatic disorder in individuals over 50 years of age and is characterized by the abrupt onset of bilateral pain and prolonged stiffness involving the shoulder and pelvic girdles (1). Corticosteroids are the cornerstone of therapy for PMR, with most patients exhibiting a rapid and marked clinical response. Treatment is typically initiated with prednisone at 10–15 mg/day, followed by gradual tapering, with discontinuation generally achieved within 12–18 months of disease onset. Nevertheless, up to 50% of patients develop corticosteroid dependence defined as the inability to taper or discontinue treatment without relapse or worsening of disease activity. In such cases, as well as in patients at high risk of adverse events related to prolonged corticosteroid use, the addition of a corticosteroid-sparing agent is warranted (2). Methotrexate has been the most prescribed disease-modifying antirheumatic drug for these patients, however, its efficacy in PMR remains controversial (3, 4).

Tocilizumab, a human monoclonal antibody targeting the interleukin-6 receptor (IL-6R), has been evaluated in the phase 2/3 *Polymyalgia Rheumatica Glucocorticoid-Sparing (PMR-SPARE)* trial, which enrolled patients with newly diagnosed PMR and implemented a rapid corticosteroid-tapering regimen. More than 50% of the patients treated with tocilizumab achieved corticosteroid-free remission at weeks 16 and 24, compared with 20% in the placebo group. In addition, tocilizumab was associated with a reduced relapse rate and an approximately 40% decrease in cumulative corticosteroid exposure (5). More recently, sarilumab, another human monoclonal antibody directed against IL-6R, was approved for PMR on the basis of the *Sarilumab in Patients with Polymyalgia Rheumatica (SAPHYR)* trial. This study included patients who experienced disease flares during corticosteroid tapering and demonstrated that those receiving sarilumab were more likely to achieve sustained remission at 1 year and had a lower cumulative corticosteroid exposure compared with placebo. These effects were observed independently of changes in C-reactive protein (CRP) levels (6).

Accordingly, IL-6 inhibitors are currently considered a therapeutic option for patients with corticosteroid-dependent PMR or those at high risk of glucocorticoid-related adverse effects. Interleukin-6 is a key regulator of hepatic acute-phase reactant (APR) production. The IL-6R is expressed on a limited range of cell types, including hepatocytes. Binding of IL-6 to IL-6R induces dimerization of the signal-transducing subunit gp130, thereby activating intracellular signaling cascades, including the signal transducer and activator of transcription 3 (STAT3) pathway, which ultimately drives the expression of acute-phase response genes. Experimental studies in murine models have shown that IL-6 deficiency leads to an attenuated acute-phase response following inflammatory stimuli, underscoring its central role in hepatic APR induction (7). Consequently, pharmacologic blockade of IL-6 signaling suppresses APR production. In patients initiating treatment with tocilizumab or sarilumab, CRP and erythrocyte sedimentation rate (ESR) levels decline rapidly, often preceding clinically apparent improvement. This dissociation may result in a discrepancy between true disease activity and conventional inflammatory markers.

Consequently, the validity of assessment tools based on APRs has been questioned, as they may overestimate the treatment effects of IL-6–targeted therapies (8).

A disease-specific activity index, the PMR Activity Score (PMR-AS), and its imputed version (PMR-ASimp), in which CRP is replaced by a value estimated from the remaining score components, are widely used to assess disease activity. Both indices incorporate subjective patient-reported outcomes as well as CRP levels, the latter being directly influenced by IL-6-targeted therapies (9, 10). Moreover, the high burden of age-related degenerative comorbidities in patients with PMR may further hinder accurate attribution of pain to active inflammatory disease. Consequently, the objective assessment of disease activity in patients with PMR treated with anti–IL-6 therapy remains challenging, underscoring the need for novel and reliable biomarkers to accurately monitor inflammatory activity in this population.

Increasing evidence suggests that choroidal thickness (CT), measured by optical coherence tomography (OCT), may serve as a novel biomarker of systemic inflammatory diseases (11). Overall, CT appears to increase during the acute phase of inflammatory conditions and decrease following treatment response and disease remission (12–14). In a recent pilot study, our group evaluated changes in CT in patients with newly diagnosed PMR after initiating corticosteroid therapy. We found that CT was increased at diagnosis and during periods of high disease activity, as assessed by clinical indices, and decreased following corticosteroid treatment and disease remission. We also observed a high concordance between CT and CRP. These findings suggest that CT may represent a potential biomarker of systemic inflammation in patients with PMR (15).

Therefore, the aim of this study was to evaluate changes in CT in patients with corticosteroid-dependent PMR at baseline and at 1 and 6 months after initiation of anti–IL-6 therapy. Secondary objectives were to assess the correlations between CT or CRP and other clinical parameters at each follow-up visit; to evaluate the correlation between changes in these variables over time; and to determine the level of agreement between changes in CT and CRP, as well as between CT and the PMR-AS throughout follow-up.

## 2 Materials and methods

### 2.1 Participants

This observational, prospective, descriptive, longitudinal pilot study included 24 patients with corticosteroid-dependent PMR and those at high risk of corticosteroid-related adverse effects.

Participants were recruited from the Rheumatology Department of University Hospital Infanta Sofía, a secondary-level hospital in Madrid, Spain. All patients were aged ≥18 years, fulfilled the classification criteria for PMR, and were candidates for anti–IL-6 therapy (16). Corticosteroid dependence was defined as the occurrence of disease flare during corticosteroid tapering (17). Exclusion criteria included a history of inflammatory ocular disease, malignancy within the previous 5 years, recurrent infections, diverticulitis, intestinal perforation, and pregnancy. Patients with suspected giant cell arteritis (GCA) based on suggestive symptoms and comprehensive clinical and laboratory evaluation, were also excluded.

The study was conducted in accordance with the Declaration of Helsinki. Written informed consent was obtained from all participants. The study was approved by the institutional review board (approval code: A06/24).

### 2.2 Examination protocol

**Figure 1:**
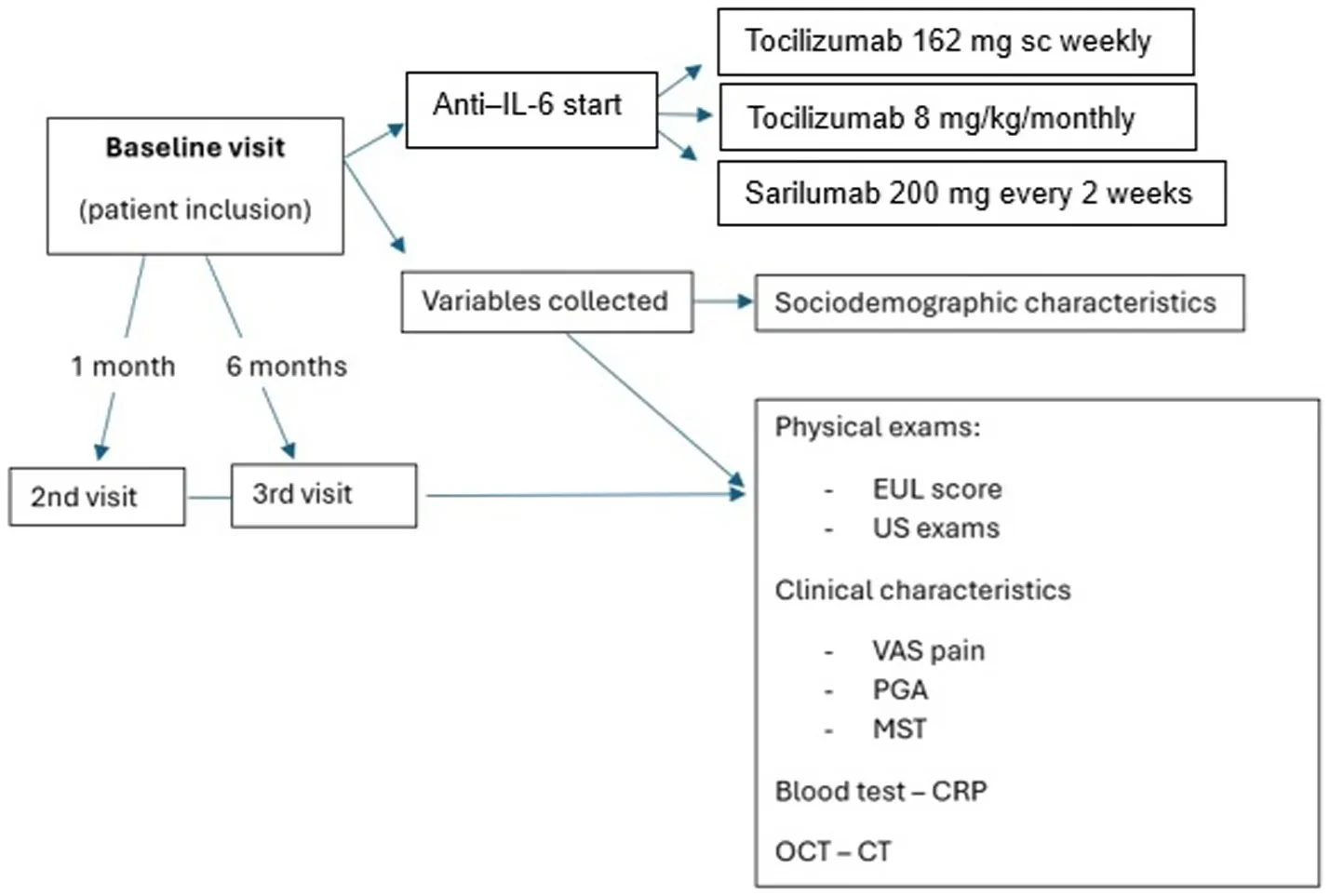
Study flowchart.

Patients attended three study visits (Figure 1). The baseline corresponded to the initiation of anti–IL-6 therapy. At baseline, patients received either subcutaneous tocilizumab (162 mg weekly), intravenous tocilizumab (8 mg/kg monthly), or subcutaneous sarilumab (200 mg every 2 weeks). The choice of agent and route of administration was left to the discretion of the treating physician. Follow-up visits were performed at 1 and 6 months after treatment initiation. The clinical data collected and the complementary assessments were identical to those obtained in our previously reported cohort of corticosteroid-treated patients with PMR: prednisone dose, CRP values, pain intensity assessed using a visual analgue scale (VAS pain), physician global assessment (PGA), duration of morning stiffness (MST), PMR-AS and PMR-ASimp scores, patients’ ability to elevate the upper limbs (EUL). Ultrasound (US) examination of shoulders and hips was also performed to assess for the presence of bicipital tenosynovitis (BT), subacromial-subdeltoid (SASD) bursitis, trochanteric bursitis, and hip synovitis (15).

The PMR-AS was calculated according to the following formula: PMR-AS = CRP (mg/dL) + VAS pain (0–10) + PGA (0–10) + (MST [min] × 0.1) + EUL (3–0) (18). The PMR-ASimp was a modified version of the PMR-AS in which CRP was replaced by a value estimated from the remaining score components. PMR-ASimp was calculated using the following equation (19):


1.12(clin−PMR−AS)+0.26

In this equation, clinPMR-AS represents the sum of MST × 0.1, EUL, VAS pain score, and PGA. PMR-AS values were interpreted as follows: scores ≤1.5 indicated remission, whereas scores >1.5 to <7, 7–17, and >17 corresponded to low, moderate, and high disease activity, respectively (9).

OCT images were acquired by a trained optometrist using a swept-source OCT (Topcon DRI-OCT Triton), and CT measurements were performed by an ophthalmologist with expertise in the field. OCT is a non-invasive imaging technique that does not involve ionizing radiation, is easy to perform, and does not require mydriatic agents. Swept-source OCT uses longer-wavelength light, enabling deeper tissue penetration and improved visualization of ocular structures. A key advantage of this technique is its ability to provide automated measurements of CT. Subfoveal CT was defined as the distance between the outer reflective border of the retinal pigment epithelium and the inner boundary of the choroid-sclera junction, identified as a hyperreflective interface. A common methodological consideration in studies assessing measurement precision is whether to include data from one or both eyes of each participant, given that measurements from fellow eyes are typically highly correlated. Two principal approaches have been proposed: analyzing a single eye per participant or including both eyes while statistically accounting for the inter-eye correlation (20). Ruiz-Medrano et al. reported a thicker nasal macular choroid in the right eye than in the left eye, whereas no significant differences were observed between the eyes for subfoveal or temporal CT (21). Similarly, Kim et al. found no statistically significant differences in CT between eyes across corresponding regions. Based on these findings, and given that subfoveal CT was measured, the eye with the greater CT value was selected for analysis in our study, although measurements were obtained in both eyes of each participant (22).

### 2.3 Sample size

Based on clinical practice, we estimated that in patients with PMR, CT proposed as a biomarker of inflammatory activity, decreases significantly following improvement in systemic inflammation after 6 months of anti–IL-6 therapy. As no previous studies have quantified the expected magnitude of CT reduction in patients with PMR receiving anti–IL-6 therapy, a formal sample size calculation based on anticipated effect size was not feasible. Accordingly, the present study was designed as a pilot investigation with an estimated sample size of 24 patients.

### 2.4 Statistical analysis

A descriptive analysis of categorical variables was performed using absolute (n) and relative (%) frequencies. The Shapiro–Wilk test was performed to assess the normality of data. Quantitative variables are presented as mean ± standard deviation (SD) for normally distributed data and as median with interquartile range [IQR] for non-normally distributed data.

Changes in CT and other clinical parameters over time (baseline, 1 month, and 6 months) were assessed using paired-sample comparisons based on the *t* test or the Wilcoxon signed-rank test, as appropriate, according to the normality of the data distribution. Correlations between quantitative variables, including changes in CT and reduction in prednisone dose, as well as between CRP levels and prednisone dose, were assessed using Pearson or Spearman correlation coefficients, as appropriate based on data normality.

Agreement between CT findings and other clinical parameters (CRP, PMR-AS, and PMR-ASimp) was evaluated by calculating the percentage agreement and corresponding 95% confidence intervals (95% CIs) at each follow-up visit. Bland–Altman analyses were subsequently performed to assess the mean differences (bias) and limits of agreement between CT findings and the corresponding clinical measures.

Subgroup analyses were performed to compare clinical outcomes between patients with and without rotator cuff disease (RCD). Categorical variables were compared using the chi-square test or Fisher’s exact test, according to expected frequencies. Quantitative variables were compared using the independent-samples Student’s *t* test for normally distributed data and the Mann–Whitney *U* test for non-normally distributed data. A 2-sided *p* value < 0.05 was considered statistically significant. Statistical analyses were performed using IBM SPSS Statistics, version 25.0 (IBM Corp., Armonk, NY, USA).

## 3 Results

### 3.1 Demographic and clinical characteristics

A total of 24 patients were enrolled between March 2024 and October 2024 including 21 women and 3 men, with a mean age of 74.5 ± 5.9 years. The median interval between PMR diagnosis and initiation of anti–IL-6 therapy was 9.5 [5.25] months. Among the included patients, 21 (87.5%) met criteria for corticosteroid-dependence disease, whereas the remaining 3 patients (12.5%) received anti–IL-6 therapy because of concomitant diabetes mellitus. Sixteen patients (66.7%) initiated subcutaneous tocilizumab; 2 (8.3%) received intravenous tocilizumab, and 6 (25%) started subcutaneous sarilumab at a dose of 200 mg every 2 weeks. Comorbidities were present in 75% of patients, with dyslipidemia being the most common (45.8%), followed by arterial hypertension (37.5%) and diabetes mellitus (20.8%). The remaining baseline demographic and clinical characteristics are summarized in Tables 1, 2. At baseline, disease activity was classified as high in 19 patients (79.2%) and moderate in 5 patients (20.8%) At the 1-month follow-up visit, disease activity was high in 3 patients (12.5%), moderate in 20 (83.3%) and low in only 1 patient (4.2%). At the final follow-up visit, only 1 patient (4.2%) continued to exhibit high disease activity, whereas 9 patients (37.5%) had moderate disease activity and 9 (37.5%) had low disease activity. Remission was achieved in 5 patients (20.8%).

**Table 1:** Demographic characteristics.

Variable	Value
Age (years)	74.5 ± 5.9
Gender (women)	21 (87.5%)
Months from diagnosis and anti-IL-6 start	9.5 [25.8]
CT (μm)	214.0 ± 71.1
Prednisone dose (mg)	7.5 [5.0]
CRP (mg/dl)	0.8 [1.2]
VAS pain	8.0 [1.0]
PGA	8.0 [2.5]
MST (minutes)	120.0 [82.5]
PMR-AS	27.7 ± 7.1
PMR-ASimp	29.9 ± 7.9
DL	45.8% (IC95% 25.9–65.8)
HTA	37.5% (IC95% 18.3–56.9)
DM	20.8% (IC95% 4.6–37)

Qualitative variables are expressed as absolute frequency (percentage). Quantitative variables are expressed as mean ± SD or median [IQR]. CT, choroidal thickness; CRP, C-reactive protein; VAS, visual analog scale; PGA, physician’s global assessment; MST, morning stiffness; PMR, polymyalgia rheumatica; PMR-AS, PMR disease activity score; PMR-ASimp, imputed PMR-AS; DL, dyslipidemia; HTA, hypertension; DM, diabetes mellitus.

**Table 2:** Elevation upper limbs (EUL) baseline grades and ultrasonography (US) baseline findings.

Variable	Patients (%)
EUL grades	
Grade 0	2 (8.4%)
Grade 1	11 (45.8%)
Grade 2	11 (45.8%)
Grade 3	0 (0.0%)
US findings	
BT	15 (62.5%)
SASD bursitis	7 (29.2%)
Other findings	2 (8.3%)

Mean CT at baseline was 214 ± 71.13 μm. CT decreased significantly at 1 month (208.46 ± 70.03 μm, *p* < 0.001) and at 6 months (183.67 ± 70.37 μm, *p* < 0.001) of follow up (Table 3). A statistically significant decrease from baseline was observed at both 1 and 6 months in the following parameters: prednisone dose, CRP values, VAS pain, PGA, MST, EUL, PMR-AS, and PMR-ASimp (all *p* < 0.001) (Table 3). BT was the most frequent US finding, being detected in 15 patients (62.5%) at baseline. The prevalence of BT decreased to 9 patients (37.5%) at 1 month and to 4 patients (16.7%) at 6 months. Regarding changes in CT according to the type of anti–IL-6 therapy, no statistically significant differences were observed between patients treated with tocilizumab and those receiving sarilumab at baseline (*p* = 0.384), 1 month (*p* = 0.548), or 6 months (*p* = 0.483) (Table 4).

**Table 3:** Evolution of variables along study visits.

Variable	Basal	First month	p*	Sixth month	p**	p***
CT	214 ± 71.1	208.5 ± 70	<0.001	183 ± 70.4	<0.001	<0.001
Prednisone dose	7.5 (5)	5 (2.5)	<0.001	2.5 (2.2)	<0.001	<0.001
CRP	0.8 (1.2)	0.1 (0.1)	<0.001	0.1 (0.1)	<0.001	0.950
VAS pain	8 (1)	5 (1)	<0.001	2 (3.8)	<0.001	<0.001
PGA	8 (2.5)	4.5 (1)	<0.001	1.5 (3.8)	<0.001	<0.001
MST	120 (82.5)	25 (28.8)	<0.001	5 (10)	<0.001	0.004
PMR-AS	27.7 ± 7.1	13.1 ± 4.2	<0.001	7 ± 5.3	<0.001	<0.001
PMR-AS clin	26.5 ± 7.1	13 ± 4.2	<0.001	6.8 ± 5.3	<0.001	<0.001
PMR-AS imp	31.6 (10.6)	14.8 (4.2)	<0.001	6.1 (8.8)	<0.001	0.024

*Comparison between baseline and 1 month, **Comparison between baseline and 6 months, ***Comparison between 1 month and 6 months. CT, choroidal thickness; CRP, C-reactive protein; VAS, visual analog scale; PGA, physician’s global assessment; MST, morning stiffness; EUL, elevate the upper limbs; PMR, polymyalgia rheumatica; PMR-AS, PMR disease activity score; PMR-ASimp, imputed PMR-AS.

**Table 4:** Comparatives between type of IL-6.

*T*	Tocilizumab	Sarilumab	*p* value
Baseline to 1 month			
CT	3.5 [6]	6.5 [5.5]	0.384
CRP	0.7 [1]	1.1 [4.6]	0.526
Dose of prednisone	2.5 [5]	0.6 [2.5]	0.097
VAS pain	14.0	4.0	0.618
PGA	4.0 [2.5]	2.5 [3]	**0.029**
MST	74.4 ± 48.8	55.8 ± 37.2	0.404
EUL	0.0 [1]	1.0 [0.5]	**0.038**
PMR-AS	15.2 ± 7.9	12.6 ± 6.1	0.464
PMR-Asimp	16.2 ± 8.8	11.9 ± 5.9	0.275
Baseline to 6 months			
CT	23 [30]	18 [41.3]	0.548
CRP	0.8 [1]	1 [4.2]	0.714
Dose of prednisone	5 [4.1]	2.5 [3.8]	**0.038**
VAS pain	16	5	1.000
PGA	7 [4.3]	2.5 [3.5]	**0.028**
MST	90.6 ± 60.2	67.5 ± 47.8	0.405
EUL	1.0 [1]	1.0 [1.3]	0.241
PMR-AS	22.2 ± 12	16.2 ± 8.6	0.273
PMR-Asimp	24 ± 13.1	16.1 ± 10.2	0.392
1 month to 6 months			
CT	18.5 [22.3]	11 [37]	0.483
CRP	0 [0]	−0.1 [0.4]	0.067
Dose of prednisone	2.5 [2.8]	0.6 [3.1]	0.089
VAS pain	14	2	0.129
PGA	3 [3.3]	0 [3.5]	0.125
MST	20 ± 26.3	0 ± 27.5	0.180
EUL	0 [1]	0 [0.3]	0.594
PMR-AS	9.1 ± 8.6	0.1 ± 8.7	0.243
PMR-Asimp	10.4 ± 9.7	0.8 ± 9.7	0.270

CT, choroidal thickness; CRP, C-reactive protein; VAS, visual analog scale; PGA, physician’s global assessment; MST, morning stiffness; EUL, elevate the upper limbs; PMR, polymyalgia rheumatica; PMR-AS, PMR disease activity score; PMR-ASimp, imputed PMR-AS. Values expressed as means and standard deviation in brackets.

### 3.2 Correlations and level of agreement

A significant correlation was observed between the decrease in CT and the reduction in prednisone dose from month 1 to month 6 of follow-up (*r* = 0.417, 95% CI = 0.003–0.708; *p* = 0.043). In contrast, no significant correlations were found between changes in CT and the remaining variables during follow-up. A statistically significant correlation was observed between CRP levels and prednisone dose at 6 months (*r* = 0.452, 95%CI = 0.048–0.730; *p* = 0.026). However, no significant correlations were observed between the other parameters at baseline, 1 month, or 6 months. Likewise, CT was not significantly correlated with any of the evaluated parameters at any study visit (Supplementary Tables S1–S4).

**Figure 2:**
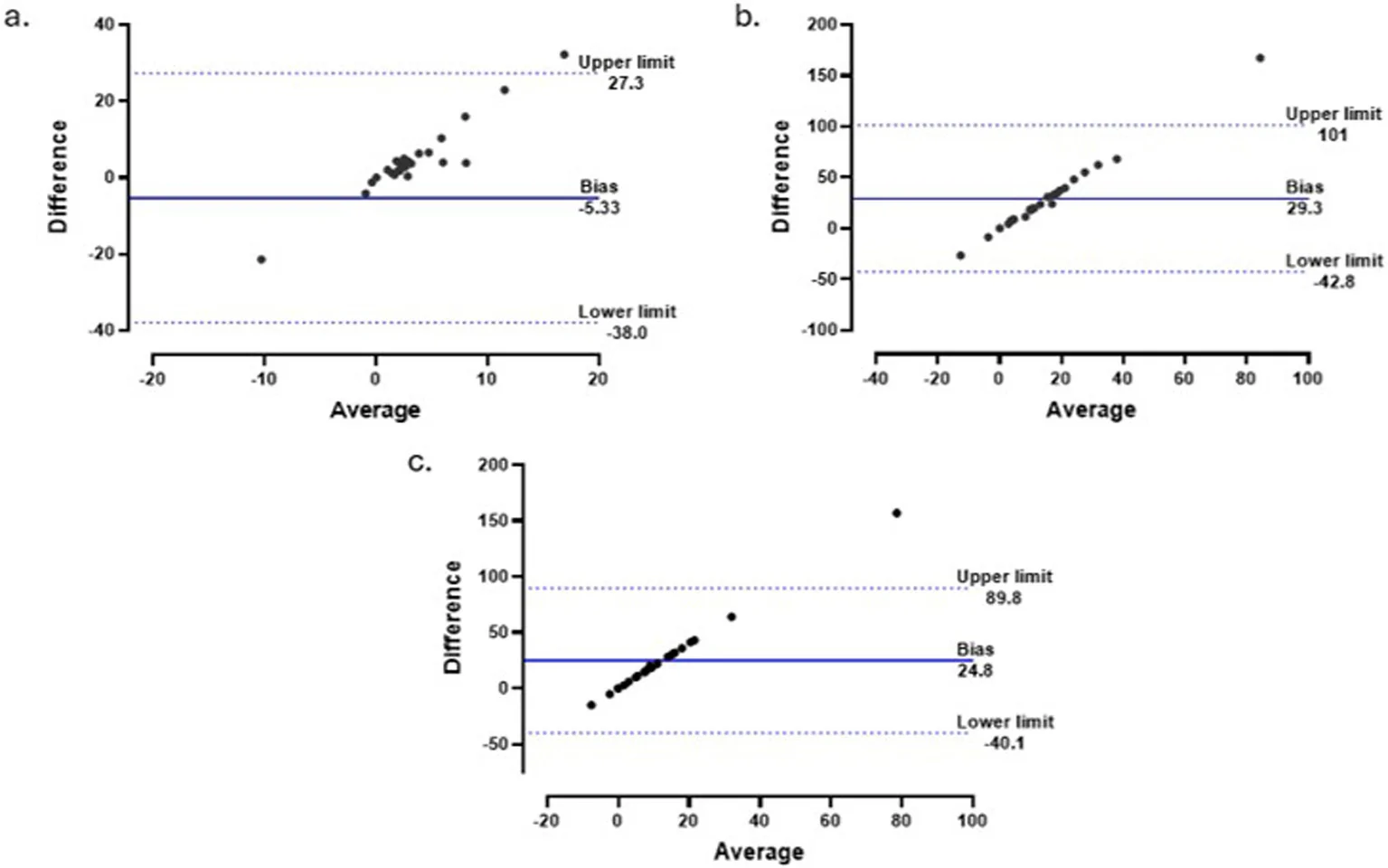
Bland–Altman plot showing the difference between CT and CRP against their mean from baseline to 1 month **(a)**, baseline to 6 months **(b)**, and 1 month to 6 months **(c)**. Most observations fall within these limits, indicating good agreement between the two methods.

Changes in CT and CRP were concordant in 20 of 24 patients (83.3%; 95% CI, 62.6–95.3%) from baseline to 1 month, a proportion significantly greater than expected by chance (exact binomial test, *p* < 0.001). Concordance remained high from baseline to 6 months (79.1%; 95% CI, 61.1–97.2%; *p* = 0.006) but decreased to 66.7% (95% CI, 44.7–84.4%; *p* = 0.152) when changes between the 1-month and 6-month visits were evaluated (Figure 2).

**Figure 3:**
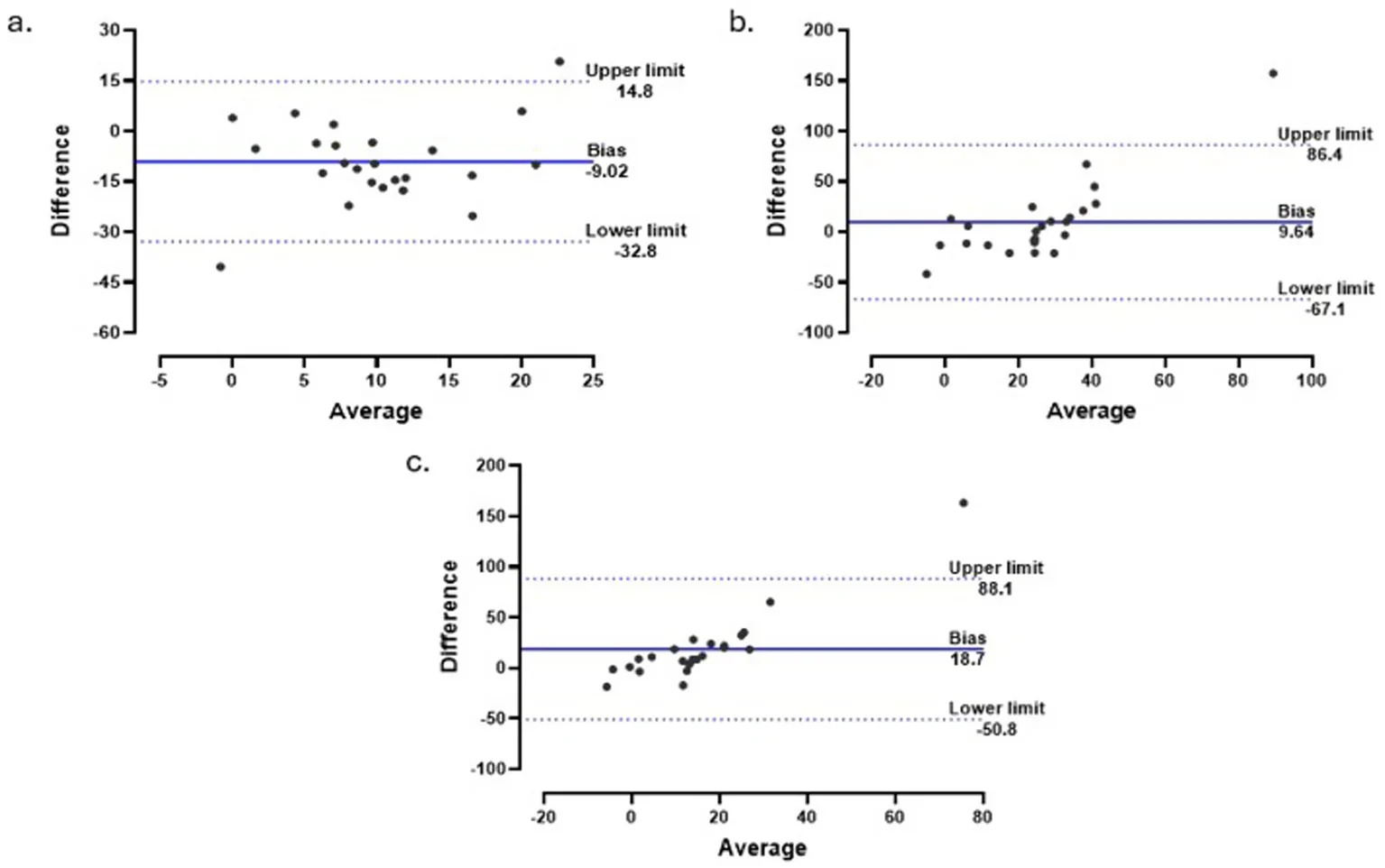
Bland–Altman plot showing the difference between CT and PMR-AS against their mean from baseline to 1 month **(a)** baseline to 6 months **(b)** and 1 month to 6 months **(c)**. Most observations fall within these limits, indicating good agreement between the two methods.

**Figure 4:**
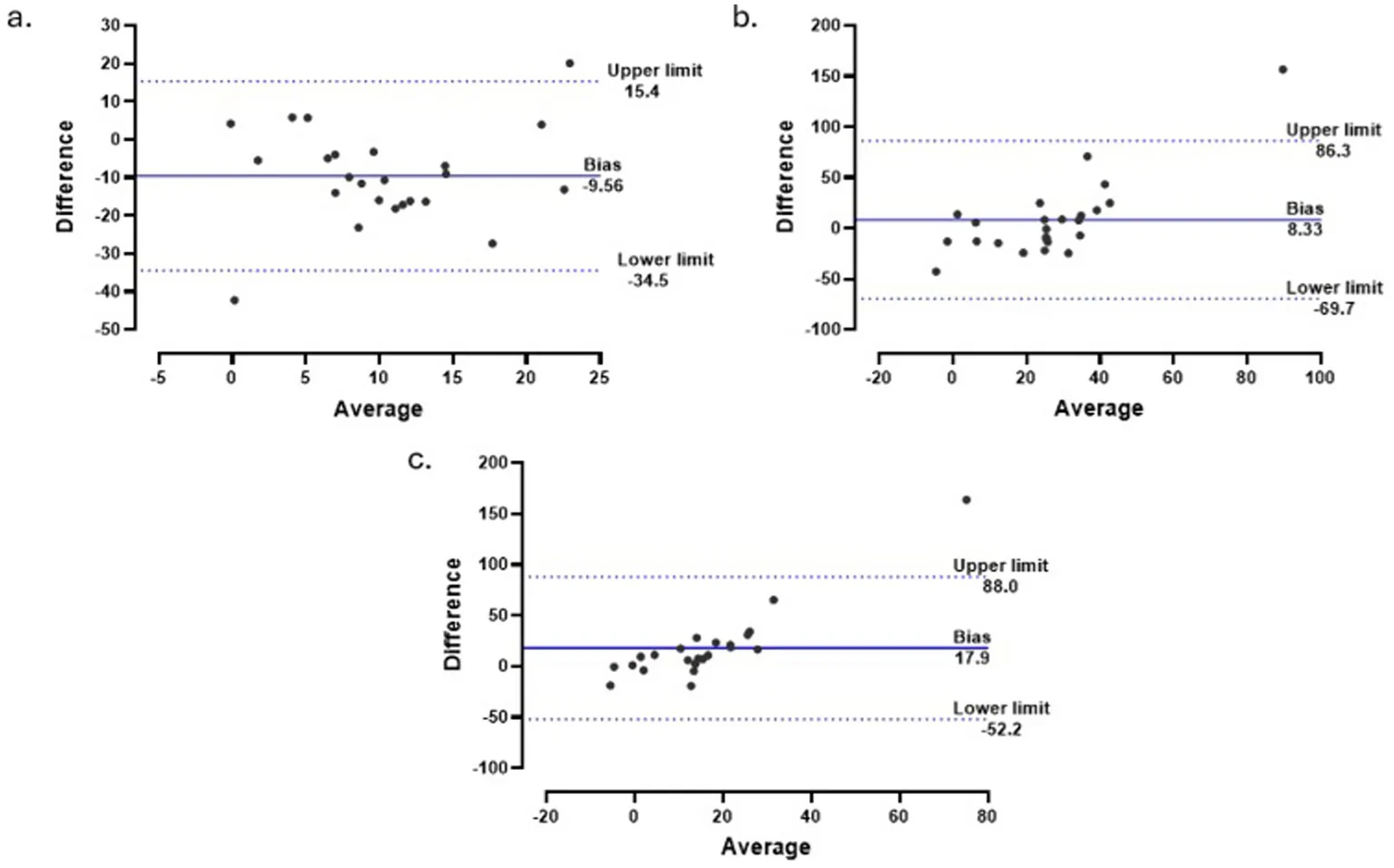
Bland–Altman plot showing the difference between CT and PMR-ASimp against their mean from baseline to 1 month **(a)** baseline to 6 months **(b)** and 1 month to 6 months **(c)**. Most observations fall within these limits, indicating good agreement between the two methods.

**Figure 5:**
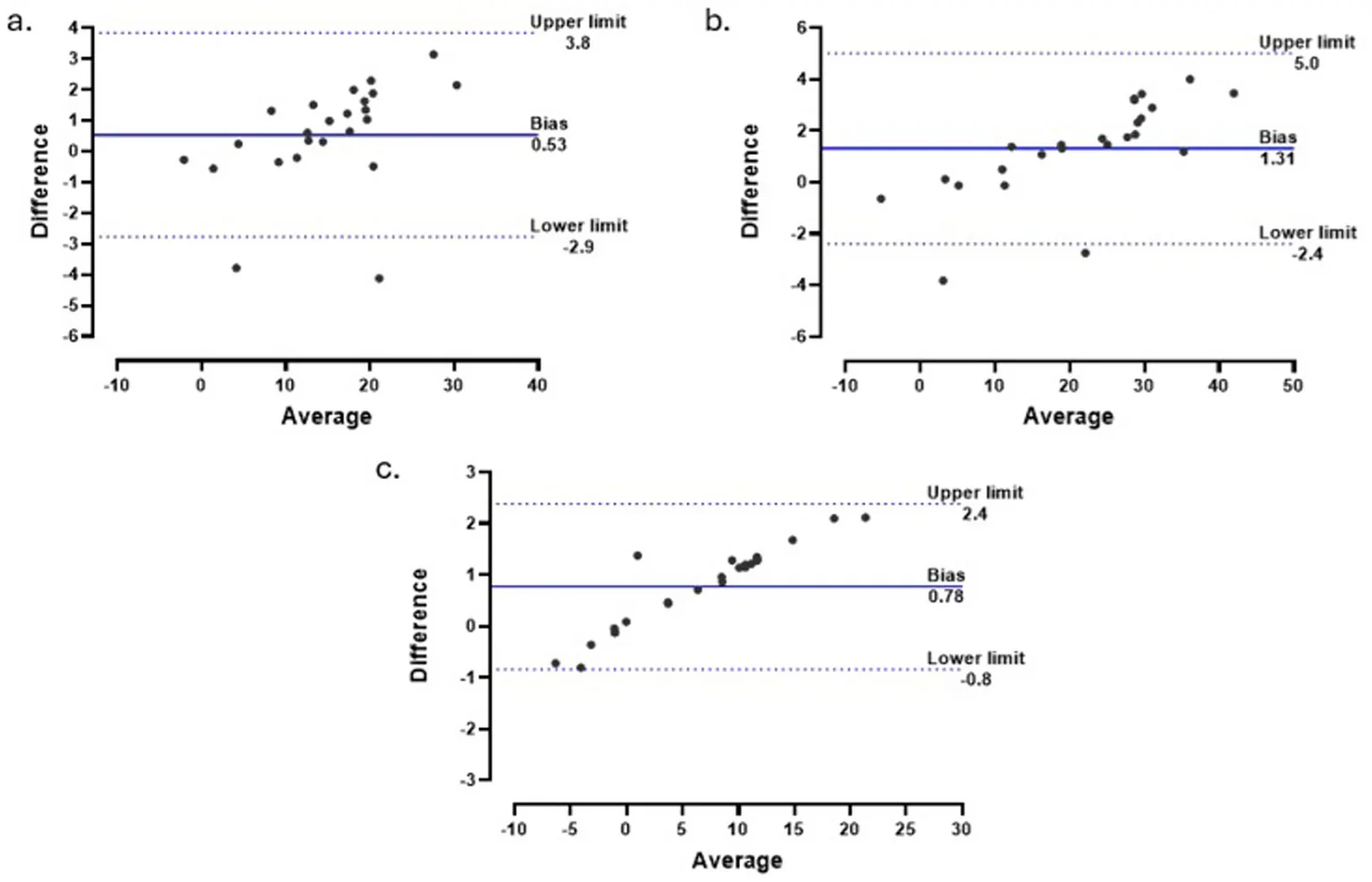
Bland–Altman plot showing the difference between PMR-AS and PMR-ASimp against their mean from baseline to 1 month **(a)**, baseline to 6 months **(b)**, and 1 month to 6 months **(c)**. All observations fall within these limits, indicating good agreement between the two methods.

The agreement between changes in CT and PMR-AS was 83.3% (95% CI, 65.8–100.0%; *p* < 0.001) from baseline to 1 month and remained unchanged from baseline to 6 months. However, agreement decreased to 75.0% (95% CI, 56.5–100.0%; *p* = 0.011) when changes between the 1-month and 6-month visits were compared (Figure 3). Similar levels of agreement were observed between changes in CT and PMR-ASimp (Figure 4). Agreement between PMR-AS and PMR-ASimp was 100% (95% CI, 88.3–100.0%; *p* < 0.001) across all study intervals (Figure 5).

Thirteen patients had concomitant rotator cuff disease (RCD). No significant differences were observed between patients with and without RCD in the proportion showing improvement in VAS pain across any of the three study intervals (baseline to 1 month, baseline to 6 months, and 1 month to 6 months), with improvement rates of 76.9% versus 72.7% (*p* = 1.00), 84.6% versus 90.9% (*p* = 1.00), and 61.5% versus 72.7% (*p* = 0.679), respectively. Similarly, among the 13 patients with RCD, improvement in BT was observed throughout follow-up; however, these changes did not reach statistical significance across the evaluated intervals (*p* = 0.649 baseline-1 month; *p* = 0.973 baseline-6 months and *p* = 0.973 1 month-6 months).

## 4 Discussion

In this study, CT findings decreased significantly in patients with active PMR following initiation of anti–IL-6 therapy. Furthermore, changes in CT showed a high level of agreement with changes in disease activity indices.

Compared with our previous study, which included only corticosteroid treatment and assessed CT for the first time 3 months after treatment initiation, the present study evaluated CT changes as early as 1 month after the initiation of anti–IL-6 therapy (15). Because IL-6 blockade is expected to suppress inflammation more rapidly than glucocorticoid therapy alone, evaluating CT as early as 1 month after treatment initiation provides a more stringent assessment of its responsiveness as an imaging biomarker. Although inflammation of large vessels may persist despite clinical remission, the choroid, a highly vascularized microvascular tissue, may respond more rapidly to the resolution of systemic inflammation, resulting in earlier structural changes detectable by OCT (23).

CT is altered in systemic inflammatory diseases because the choroid has the highest blood flow per unit tissue weight in the human body, making it particularly susceptible to inflammation-induced vascular changes (11).

In our previous study, CT was increased in patients with active PMR and decreased following corticosteroid therapy in parallel with inflammatory markers, suggesting that choroidal thickening is primarily driven by inflammatory activity rather than primary vascular damage (15). Given the central role of IL-6 in PMR pathogenesis and the rapid clinical response to IL-6 blockade, this cytokine may contribute, directly or indirectly, to the observed changes in CT. However, additional vascular mediators are also likely to be involved. Increased circulating levels of vascular endothelial growth factor (VEGF) and angiopoietin-2 (Ang-2) have been reported in active PMR, supporting the presence of endothelial activation, increased vascular permeability, and vascular remodelling (24).

Van Sleen et al. reported that all angiogenesis-related markers, with the exception of angiopoietin-1, were elevated in patients with PMR compared with healthy controls. Higher Ang-2 levels were associated with prolonged glucocorticoid requirements and a less favourable disease course. In addition, baseline Ang-2 assessment may help identify patients with concomitant vasculitis. These findings suggest that Ang-2 may represent a promising diagnostic and prognostic biomarker in PMR (25).

Although direct evidence linking these pathways to changes in CT in PMR is currently lacking, it is plausible that choroidal thickening reflects the combined effects of systemic inflammation and secondary microvascular dysfunction. Future studies integrating OCT-derived CT measurements with circulating inflammatory and vascular biomarkers are warranted to elucidate the mechanisms underlying these changes.

CT measurement by OCT is a simple, easily performed technique that is widely available in most ophthalmology departments. Its noninvasive nature and rapid acquisition represent clear advantages over alternative methods for assessing disease activity, such as positron emission tomography–computed tomography (PET-CT), which is associated with radiation exposure, longer acquisition times, and more limited availability across healthcare settings. CT is influenced by multiple factors, with age being one of the main determinants. Several studies have shown that CT is thinner in older individuals, and some have reported an average decrease of approximately 15 μm per decade of life (26–28).

Wakatsuki et al. evaluated CT across different age groups and found that in individuals aged 60–79 years, mean CT was 179.2 ± 48.8 μm (29). In our cohort (mean age, 74 years), CT values at 6 months after initiation of anti–IL-6 therapy were comparable to age-matched reference values, suggesting that the observed changes are unlikely to be attributable to aging alone.

Our findings support the potential role of CT as a biomarker of systemic inflammation and treatment response. However, interindividual variability and other contributing factors should be considered when interpreting longitudinal changes. CT can also vary according to sex; however, sex-based differences were not evaluated in our study, as the cohort was predominantly women. The timing of CT assessment is also relevant, given the influence of circadian variation. The choroid is typically thicker in the early morning, with a gradual decrease observed until approximately 5 p.m. (30, 31). To minimize measurement bias, all OCT scans were performed between 9:00 and 11:00 a.m. Comorbidities may also affect the choroid. Patients with arterial hypertension or dyslipidemia tend to have a thicker choroid, whereas findings in diabetes mellitus are inconsistent (26). Since most patients with PMR are of an age at which comorbidities that may influence CT are common, comparisons of absolute CT values between patients are challenging. For this reason, we focused on within-patient changes in CT over the 6-month follow-up, rather than between-patient comparisons, given that age, comorbidities, and concomitant treatments may act as potential confounders. CT measurement may also be influenced by administered treatments. Ferreria et al. reported that patients receiving higher doses of corticosteroids exhibited increased CT values (32). In contrast, Han et al. evaluated the effect of corticosteroid therapy on CT in patients with systemic diseases and found no significant impact of treatment on CT (33). Similarly, a pilot study in patients with PMR receiving corticosteroids reported no correlation between corticosteroid dose and CT, suggesting that corticosteroid therapy does not substantially influence CT in this population (15).

Therefore, in our study, baseline CT measurements may reflect underlying disease activity in patients who had received corticosteroids for several months prior to initiation of anti–IL-6 therapy. Moreover, the subsequent decrease in CT appears to be associated with effective control of inflammation achieved with anti–IL-6 therapy rather than with the effects of concomitant corticosteroid treatment. All patients in our cohort had received corticosteroid therapy prior to initiation of anti–IL-6 therapy, which may explain the lower CRP levels observed at the baseline visit compared with those typically expected in newly diagnosed, treatment-naïve patients at disease onset. Both IL-6 inhibitors, tocilizumab and sarilumab, target the IL-6R, although they differ in their binding affinity. Sarilumab has been reported showed to exhibit greater affinity than tocilizumab, resulting in more potent inhibition of IL-6 signaling and downstream cellular responses (34). In the present study, patients received either sarilumab or tocilizumab at the discretion of the treating physician. No statistically significant differences were observed between the two agents in CT or in most of the evaluated parameters. However, PGA values at 1 and 6 months, as well as prednisone dose at 6 months, were significantly lower in patients receiving sarilumab. This finding may be related to the higher receptor affinity of sarilumab, potentially leading to earlier clinical improvement and facilitating a greater reduction in corticosteroid exposure. The absence of significant differences in the remaining parameters may reflect the limited sample size and warrants further evaluation in larger cohorts. Previous studies and clinical trials of tocilizumab have shown comparable efficacy between intravenous and subcutaneous formulations. Accordingly, the route of administration is unlikely to have substantially influenced changes in inflammatory parameters in our cohort. Nevertheless, only 2 patients received intravenous therapy, and no subgroup analyses were performed in this population. We observed significant correlations between the reduction in CT and prednisone dose over time, as well as between CRP levels and prednisone dose at 6 months, supporting the role of both CT and CRP as markers of treatment response in this population. By month 6, most patients had transitioned from moderate or high disease activity to low disease activity or remission, allowing substantial corticosteroid tapering. Only three patients continued to require prednisone doses >5 mg/day. However, we did not find any correlation between CT or CRP and the other parameters, either at individual visits or in changes over the course of follow-up. Consistent with previous reports, the lack of correlation may be attributable not only to the limited sample size but also to the comparison of objective and subjective outcome measures (15, 35). In addition, direct comparisons across variables may be challenging due to substantial differences in measurement scales and units (for example, CT is expressed in μm, whereas MST is measured in minutes).

The high level of agreement between changes in CT and CRP, as well as between CT and PMR-AS, from baseline to the 1-month and 6-month visits suggests that these measures may offer complementary insights into disease activity. Accordingly, CT may represent a useful alternative biomarker when CRP measurements are unavailable or when PMR-AS cannot be calculated because of missing CRP values. As expected, agreement was lower when changes between the 1-month and 6-month visits were compared, likely reflecting the rapid suppression of CRP following initiation of anti–IL-6 therapy, which frequently results in very low or undetectable CRP levels as early as the first month of follow-up.

The high level of agreement between PMR-AS and PMR-ASimp was expected, as both indices share the same underlying structure and PMR-ASimp is directly derived from PMR-AS. Our findings are in line with those of D’Agostino et al., who reported consistently high concordance and negligible longitudinal differences among disease activity measures, including the imputed version (36). These observations support the use of PMR-AS and PMR-ASimp as interchangeable instruments for the assessment of overall disease activity. Accordingly, CT demonstrated an essentially identical level of agreement with both indices.

Assessment of CT during active disease and again after remission may help establish individual reference values corresponding to different disease states. In patients in remission who subsequently experience recurrent pain or worsening constitutional symptoms, repeat CT evaluation may provide valuable additional information, particularly when concomitant degenerative musculoskeletal conditions are present. If CT values remain within the range previously associated with remission, alternative explanations for the clinical deterioration should be explored rather than attributing symptoms to recurrent PMR activity. Under these circumstances, treatment intensification may not be justified, and therapeutic failure should not be inferred in patients receiving standard-of-care therapy.

BT was the most frequent US finding in our cohort. This observation differs from previous reports identifying SASD bursitis as the hallmark imaging feature of PMR (37). Huwart et al. assessed the longitudinal evolution of shoulder and hip bursitis and synovitis in patients with PMR receiving tocilizumab and demonstrated a reduction in SASD bursitis after 12 weeks of treatment (38). Unlike the corticosteroid-naïve patients included in their study, patients in our cohort had been exposed to corticosteroids before enrolment, a factor that may have contributed to the lower prevalence of SASD bursitis observed in our series. Moreover, the authors showed that clinical improvement preceded imaging remission. In line with these observations, Palard-Novello et al. reported a reduction in BT uptake on PET-CT following three doses of tocilizumab in patients with PMR, although BT changes did not correlate with other inflammatory markers (39). Similarly, in our cohort, no association was observed between clinical improvement and BT. However, the extent of BT improvement was comparable in patients with and without RCD, suggesting that BT primarily reflects inflammatory disease activity rather than structural musculoskeletal damage.

### 4.1 Limitations

The main limitation of this study is its relatively small sample size, which may have reduced statistical power and limited the generalizability of our findings. Although PMR does not directly involve ocular structures, concomitant GCA may be present in a subset of patients and can affect the ophthalmic artery, thereby representing a potential confounder in the assessment of CT. While patients with clinical features or examination findings suggestive of GCA were excluded, the possibility of subclinical extracranial large-vessel involvement cannot be completely excluded. PET-CT is currently considered the most sensitive imaging modality for identifying extracranial GCA; however, it was not incorporated into the study protocol because of its cost, limited availability, and radiation burden.

The lack of a matched healthy control group is another limitation of the present study. Nevertheless, the selection of an appropriate control population is inherently challenging in this context, as CT is influenced by numerous age-related factors and comorbid conditions that are highly prevalent in patients with PMR and difficult to balance adequately between groups. As a result, residual confounding would likely persist despite careful matching. From this perspective, a longitudinal within subject design may be methodologically preferable, as it enables each participant to serve as their own control and thereby reduces the influence of interindividual variability and comorbidity related determinants of CT.

As reported by Devauchelle-Pensec et al. ESR-based PMR-AS may serve as an alternative when CRP values are unavailable. A plausible explanation lies in the distinct effects of IL-6 blockade on APR. Whereas IL-6 is a major driver of hepatic CRP production, ESR is largely influenced by fibrinogen levels, which are regulated by a broader network of inflammatory mediators beyond IL-6 (19). As a result, anti–IL-6 therapy induces a rapid and profound suppression of CRP, while the corresponding reduction in ESR tends to be more modest. Although evaluation of the contribution of ESR would have been of considerable interest, this was not possible because ESR measurements were not available for all patients included in the analysis. Further studies in larger, well-characterized cohorts are needed to validate these findings and to more effectively account for potential sources of confounding, thereby allowing a more robust assessment of longitudinal changes in CT. Finally, the present study did not include a comparison of CT evolution in patients with PMR not receiving anti–IL-6 therapy, as this issue had already been investigated in a previous study from our group (15).

## 5 Conclusion

We found that patients with active PMR exhibited increased CT values at baseline, which decreased following the initiation of anti–IL-6 therapy in parallel with clinical improvement and reaching age normal ranges after 6 months of treatment. The strong agreement observed between longitudinal changes in CT, CRP, and PMR activity indices supports the potential utility of CT as a marker of inflammatory disease activity. CT may be particularly valuable when conventional inflammatory markers are unavailable or their interpretation is limited by IL-6 blockade. These findings warrant confirmation in larger prospective studies and further evaluation of the role of CT in the monitoring of PMR.

## Data Availability

The original contributions presented in the study are included in the article/Supplementary material, further inquiries can be directed to the corresponding author.
