# Supplementary material for: Choroidal thickness as a biomarker of inflammatory activity in patients with polymyalgia rheumatica receiving anti–IL-6 treatment
Source: Front Med (Lausanne). 2026 Aug 17;13:1917539. doi: 10.3389/fmed.2026.1917539 (PMC13527446; doi:10.3389/fmed.2026.1917539)
Supplement: Supplementary file 1 [file Data_Sheet_1.DOCX]

**Table S1. Correlation between CT and other inflammatory parameters at each visit**

| T  **Variable** | **r** | IC  **95%CI** | **p value** |
| --- | --- | --- | --- |
| **Baseline** |  |  |  |
| CRP | -0.120 | -0.509 to 0.309 | 0.576 |
| Dose of prednisone | -0.166 | -0.543 to 0.266 | 0.430 |
| VAS pain | 0.082 | -0.344 to 0.480 | 0.703 |
| PGA | -0.034 | -0.442 to 0.385 | 0.874 |
| MST | -0.165 | -0.542 to 0.267 | 0.441 |
| EUL | 0.186 | -0.247 to 0.557 | 0.385 |
| PMR-AS | -0.028 | -0.426 to 0.380 | 0.898 |
| PMR-Asimp | -0.019 | -0.419 to 0.388 | 0.931 |
|  |  |  |  |
| **1 month** |  |  |  |
| CRP | 0.085 | -0.341 to 0.482 | 0.695 |
| Dose of prednisone | -0.136 | -0.521 to 0.294 | 0.526 |
| VAS pain | 0.313 | -0.116 to 0.643 | 0.137 |
| PGA | 0.118 | -0.311 to 0.507 | 0.582 |
| MST | -0.218 | -0.579 to 0.216 | 0.307 |
| EUL | -0.202 | -0.568 to 0.231 | 0.344 |
| PMR-AS | -0.180 | -0.544 to 0.241 | 0.401 |
| PMR-Asimp | -0.183 | -0.546 to 0.238 | 0.392 |
|  |  |  |  |
| **6 months** |  |  |  |
| CRP | 0.188 | -0.245 to 0.558 | 0.379 |
| Dose of prednisone | 0.065 | -0.358 to 0.466 | 0.762 |
| VAS pain | 0.053 | -0.369 to 0.457 | 0.807 |
| PGA | 0.051 | -0.371 to 0.455 | 0.814 |
| MST | 0.094 | -0.333 to 0.489 | 0.663 |
| EUL | -0.138 | -0.522 to 0.292 | 0.519 |
| PMR-AS | -0.076 | -0.465 to 0.337 | 0.723 |
| PMR-Asimp | -0.073 | -0.462 to 0.341 | 0.736 |

*CT=choroidal thickness; CRP=C-reactive protein; VAS=visual analog scale; PGA=physician’s global assessment; MST=morning stiffness; EUL=elevate the upper limbs; PMR=polymyalgia rheumatica; PMR-AS=PMR disease activity score; PMR-ASimp=imputed PMR-AS; CI=confidence interval*

**Table S2. Correlation between changes in CT and other inflammatory parameters**

| T  **Variable** | **r** | IC  **95%CI** | **p value** |
| --- | --- | --- | --- |
| **Baseline to 1 month** |  |  |  |
| CRP | 0.079 | -0.346 to 0.478 | 0.712 |
| Dose of prednisone | -0.046 | -0.451 to 0.375 | 0.831 |
| VAS pain | 0.093 | -0.334 to 0.488 | 0.667 |
| PGA | 0.046 | -0.375 to 0.452 | 0.829 |
| MST | -0.044 | -0.449 to 0.377 | 0.839 |
| EUL | 0.172 | -0.261 to 0.547 | 0.422 |
| PMR-AS | 0.013 | -0.393 to 0.414 | 0.954 |
| PMR-Asimp | -0.001 | -0.404 to 0.403 | 0.996 |
|  |  |  |  |
| **Baseline to 6 months** |  |  |  |
| CRP | 0.294 | -0.137 to 0.631 | 0.163 |
| Dose of prednisone | 0.231 | -0.202 to 0.589 | 0.277 |
| VAS pain | 0.114 | -0.315 to 0.504 | 0.598 |
| PGA | 0.152 | -0.280 to 0.532 | 0.479 |
| MST | -0.048 | -0.453 to 0.373 | 0.824 |
| EUL | 0.087 | -0.339 to 0.484 | 0.686 |
| PMR-AS | -0.049 | -0.444 to 0.361 | 0.817 |
| PMR-Asimp | -0.064 | -0.455 to 0.349 | 0.768 |
|  |  |  |  |
| **1 month to 6 months** |  |  |  |
| CRP | -0.286 | -0.626 to 0.145 | 0.176 |
| Dose of prednisone | 0.417 | 0.003 to 0.708 | **0.043** |
| VAS pain | 0.049 | -0.372 to 0.454 | 0.819 |
| PGA | 0.070 | -0.354 to 0.470 | 0.745 |
| MST | 0.132 | -0.299 to 0.517 | 0.540 |
| EUL | -0.034 | -0.442 to 0.385 | 0.873 |
| PMR-AS | -0.243 | -0.589 to 0.178 | 0.252 |
| PMR-Asimp | -0.242 | -0.588 to 0.179 | 0.254 |

*CT=choroidal thickness; CRP=C-reactive protein; VAS=visual analog scale; PGA=physician’s global assessment; MST=morning stiffness; EUL=elevate the upper limbs; PMR=polymyalgia rheumatica; PMR-AS=PMR disease activity score; PMR-ASimp=imputed PMR-AS; CI=confidence interval*

**Table S3. Correlation between CRP and other inflammatory parameters in each visit**

| T  **Variable** | **r** | IC  **95%CI** | **p value** |
| --- | --- | --- | --- |
| **Baseline** |  |  |  |
| CT | -0.120 | -0.509 to 0.309 | 0.576 |
| Dose of prednisone | 0.300 | -0.130 to 0.635 | 0.154 |
| VAS pain | 0.067 | -0.357 to 0.468 | 0.755 |
| PGA | 0.051 | -0.371 to 0.455 | 0.813 |
| MST | 0.013 | -0.403 to 0.425 | 0.953 |
| EUL | 0.218 | -0.215 to 0.580 | 0.306 |
| PMR-AS | 0.119 | -0.299 to 0.499 | 0.580 |
| PMR-Asimp | -0.081 | -0.469 to 0.334 | 0.708 |
|  |  |  |  |
| **1 month** |  |  |  |
| CT | 0.085 | -0.341 to 0.482 | 0.695 |
| Dose of prednisone | 0.326 | -0.130 to 0.635 | 0.121 |
| VAS pain | -0.003 | -0.417 to 0.411 | 0.988 |
| PGA | -0.185 | -0.557 to 0.247 | 0.386 |
| MST | -0.128 | -0.515 to 0.302 | 0.550 |
| EUL | -0.216 | -0.578 to 0.218 | 0.311 |
| PMR-AS | 0.063 | -0.349 to 0.455 | 0.769 |
| PMR-Asimp | 0.037 | -0.372 to 0.434 | 0.864 |
|  |  |  |  |
| **6 months** |  |  |  |
| CT | 0.188 | -0.245 to 0.558 | 0.379 |
| Dose of prednisone | 0.452 | 0.048 to 0.730 | **0.026** |
| VAS pain | 0.128 | -0.301 to 0.515 | 0.550 |
| PGA | 0.172 | -0.260 to 0.547 | 0.420 |
| MST | -0.222 | -0.583 to 0.211 | 0.297 |
| EUL | -0.123 | -0.511 to 0.306 | 0.566 |
| PMR-AS | 0.160 | -0.260 to 0.529 | 0.456 |
| PMR-Asimp | 0.108 | -0.309 to 0.490 | 0.615 |

*CT=choroidal thickness; CRP=C-reactive protein; VAS=visual analog scale; PGA=physician’s global assessment; MST=morning stiffness; EUL=elevate the upper limbs; PMR=polymyalgia rheumatica; PMR-AS=PMR disease activity score; PMR-ASimp=imputed PMR-AS; CI=confidence interval*

**Table S4. Correlation between changes in CRP and other inflammatory parameters**

| T  **Variable** | **r** | IC  **95%CI** | **p value** |
| --- | --- | --- | --- |
| **Baseline to 1 month** |  |  |  |
| CT | 0.079 | -0.346 to 0.478 | 0.712 |
| Dose of prednisone | 0.050 | -0.371 to 0.455 | 0.816 |
| VAS pain | 0.038 | -0.382 to 0.445 | 0.861 |
| PGA | -0.046 | -0.451 to 0.375 | 0.831 |
| MST | -0.037 | -0.444 to 0.383 | 0.864 |
| EUL | 0.175 | -0.257 to 0.549 | 0.413 |
| PMR-AS | 0.184 | -0.237 to 0.547 | 0.391 |
| PMR-Asimp | -0.007 | -0.410 to 0.397 | 0.973 |
|  |  |  |  |
| **Baseline to 6 months** |  |  |  |
| CT | 0.079 | -0.346 to 0.478 | 0.712 |
| Dose of prednisone | 0.050 | -0.371 to 0.455 | 0.816 |
| VAS pain | 0.038 | -0.382 to 0.445 | 0.861 |
| PGA | -0.046 | -0.451 to 0.375 | 0.831 |
| MST | -0.037 | -0.444 to 0.383 | 0.864 |
| EUL | 0.175 | -0.257 to 0.549 | 0.413 |
| PMR-AS | 0.184 | -0.237 to 0.547 | 0.391 |
| PMR-Asimp | -0.007 | -0.410 to 0.397 | 0.973 |
|  |  |  |  |
| **1 month to 6 months** |  |  |  |
| CT | 0.294 | -0.137 to 0.631 | 0.163 |
| Dose of prednisone | 0.321 | -0.108 to 0.649 | 0.127 |
| VAS pain | 0.035 | -0.385 to 0.443 | 0.871 |
| PGA | 0.019 | -0.398 to 0.429 | 0.930 |
| MST | 0.060 | -0.363 to 0.462 | 0.783 |
| EUL | 0.492 | -0.098 to 0.753 | 0.015 |
| PMR-AS | 0.045 | -0.365 to 0.440 | 0.836 |
| PMR-Asimp | -0.064 | -0.455 to 0.349 | 0.768 |

*CT=choroidal thickness; CRP=C-reactive protein; VAS=visual analog scale; PGA=physician’s global assessment; MST=morning stiffness; EUL=elevate the upper limbs; PMR=polymyalgia rheumatica; PMR-AS=PMR disease activity score; PMR-ASimp=imputed PMR-AS; CI=confidence interval*
